# Two patients with Knobloch syndrome due to mutation in COL8A1 gene: case report and review of the literature

**DOI:** 10.1186/s12886-024-03418-5

**Published:** 2024-04-04

**Authors:** Tulin Aras Ogreden, Gürkan Erdoğan

**Affiliations:** 1 Medipol University, Department of Ophtalmology, Istanbul, Turkey; 2 Istanbul University Istanbul Faculty of Medicine, Istanbul, Turkey

**Keywords:** Knobloch syndrome, COL18A1 gene, Pediatric retinal detachment, Retinal surgery, Case report

## Abstract

**Background:**

Knobloch syndrome (KNO, OMIM # 267,750) is a rare ciliopathy group sydrome characterized by a collagen synthesis disorder. It represents an uncommon cause of pediatric retinal detachment. This report presents two cases with different COL18A1 gene mutations, complicated by retinal detachment.

**Case presentation:**

Both cases exhibited high myopia and various degrees of occipital skull defect. The first case, a female, had bilateral congenital retinal detachment, posterior embryotoxon, and strabismus. The second case, a male, had unilateral congenital retinal detachment and neuromotor developmental delay. The first case, diagnosed in the early months of life, underwent successful retinal reattachment surgery. However, surgery was not performed on the second case, who presented with late-stage unilateral retinal detachment and pre-phthisis.

**Conclusions:**

The report describes two patients with Knobloch syndrome, one of whom responded favorably to surgery for retinal detachment in both eyes. Successful anatomical results were achieved with early surgical interventions. It is essential to recognize the phenotypic and genetic heterogeneity within KNO.

## Case presentation

In this report, features of two cases with different COL18A1 gene mutations are presented.

### Case-1

The first case was referred to our clinic at the age of six months with a diagnosis of bilateral retinal detachment. She had a history of esotropia. The family noticed a slight color difference between the two eyes, esotropia, and an abnormality in the occipital region.

The parents had a third-degree consanguineous marriage, and the mother was undergoing neurological follow-up due to an intracranial cyst. Two siblings of the baby wore high myopic glasses, but there was no history of retinal detachment.

**Fig. 1:**
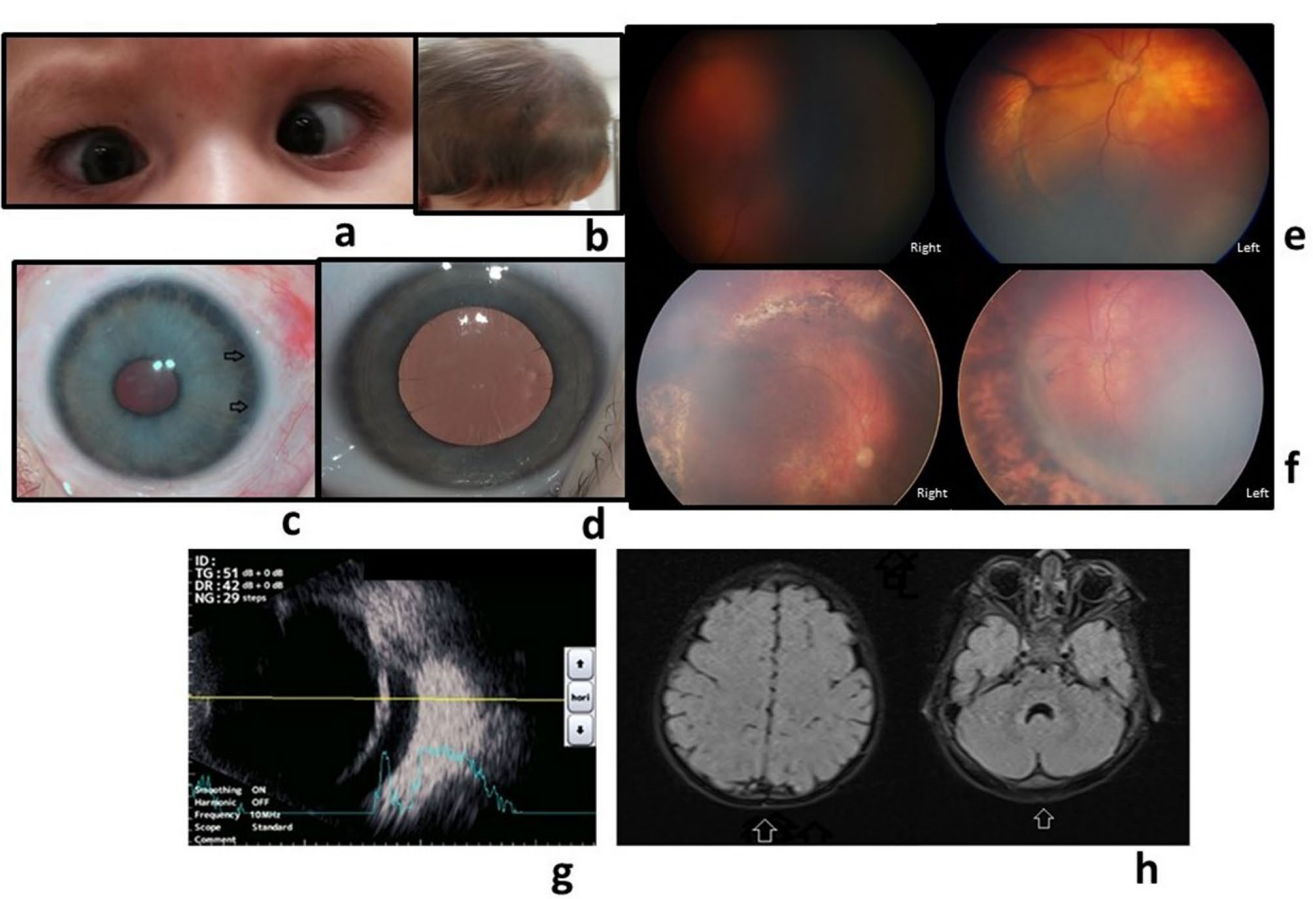
**a**-alternate esotropia **b**-occipital fat pad **c**-Right eye pupil, iris, and lens (arrows indicate posterior embryotoxon) **d**- Left eye pupil, iris and lens **e**- Fundus photo of right and left eye, chorioretinal hyperpigmented demarcation line on the peripheral retina, and shallow serous retinal detachment (The right fundus footage was not adequately illuminated due to poor pupil dilatation) **f**-Fundus views after scleral buckling, pars plana vitrectomy (PPV), and endolaser **g**- Serous detachment on B-sacan ultrasonography (USG) **h**- Cranial axial T1-weighted (T1W) magnetic resonance imaging: Occipital fat pad and midline occipital defect

The patient presented with nystagmus, doubtful object tracking, esotropia, and limitation at outward gaze (Fig. 1a). The right pupil did not dilate with medication. Refractive error was − 6.00 (Retinomax K plus5Righton, Japan) bilaterally. The skin in occipital area was hyperemic and soft on palpation (Fig. 1b).

In the right eye, a cryptless iris, iris atrophy with posterior embryotoxon, and slightly lighter iris color due to atrophy were observed (Fig. 1c, d). A paracentrally located posterior subcapsular cataract with a 1 mm diameter was also noted. Mean intraocular pressure (IOP) was 29 mmHg in the right eye and 20 mmHg in the left eye, controlled with topical medical treatment.

Ophthalmological examination in the right eye was challenging due to weak pupillary dilatation, and the fundus photograph was unclear. (PanoCam™ Wide-field Digital Imaging System Visunex, Fremont, California US)

In the left eye, a waving chorioretinal hyperpigmented demarcation line on the peripheral retina and shallow serous retinal detachment(RD) extending to the macula was observed behind it (Fig. 1e, f).

Orbital ultrasonography(USG) (UD-800, Tomey Corporation, Nagoya, Japan) revealed detachment at the posterior pole extending to the temporal and inferior quadrants in both eyes (Fig. 1g). The retinal surface was smooth, and the fluid slightly changed depending on the patient’s head position. Neither traction nor breaks were seen in the retina, and there were findings of exudative (serous) RD starting from the periphery. There was a demarcation line at the junction of the serous elevation with the normal retina, and laser could be applied on and behind this line for limitation of detachment.

Occipital fat pad and midline occipital bone defect without encephalocele were detected on magnetic resonance imaging (MRI) (Fig. 1h).

Genetic analysis revealed a mutation in the *COL18A1* gene. She had a KNO c4376C > T (p.Ser1459Leu) variant on chromosome 21.

Three hundred and sixty degrees scleral buckling surgery was performed on the right eye, followed by the other eye one week later.

The detachment in the left eye gradually decreased within 3 weeks and reattached.

At the 1-month follow-up, the retina was detached in the right eye. Due to no decrease in the height of retinal detachment in ultrasonography, pars plana vitrectomy (PPV) was performed in the right eye as the second surgery on postoperative day 34. Tamponade was not applied. The detachment gradually decreased, and the macula was reattached within two months of the second surgery in the right eye. After successful retinal reattachment in both eyes, esotropia was also corrected at the third month with bimedial recession.

### Case 2

The second case involved a 5-year-old male patient. His family observed that the right eye was smaller than the left eye. He had a diagnosis of neuromotor developmental delay and was identified with borderline macrocephaly during his neurology follow-up. The parents had a second-degree consanguineous marriage.

**Fig. 2:**
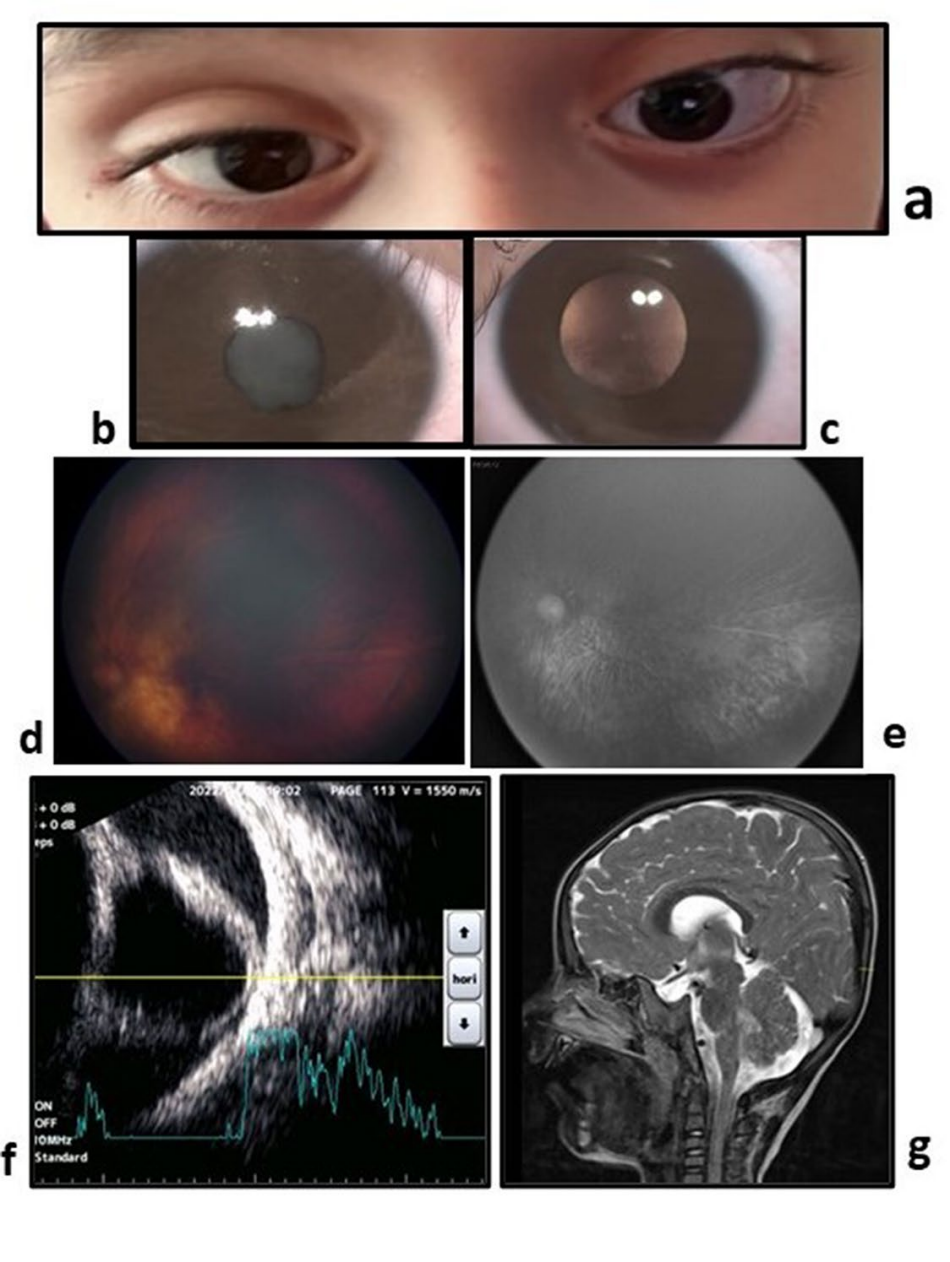
**a**- Esotropia **b**- Anterior segment photograph of the right eye, posterior synechia, and cataract **c**- The left eye, with mid-dilated pupil and clear lens **d**- fundus photo of the left eye and tessellated appearance associated with high myopia **e**- Lobular filling and attenuated vessels in FA **f**- Retinal detachment and thickening on B-scan USG **g**- Cranial Sagittal T1-weighted (T1W)MRI: Occipital fat pad and midline occipital defect

The patient’s vision was at the level of light sensation (+) in the right eye and counting fingers at 1 m in the left eye. He exhibited esotropia (Fig. 2a). Intraocular pressure (IOP) measured 12 mmHg in the right eye and 16 mmHg in the left eye. Corneal diameter was manually measured with a caliper, was 11 mm on the right and 12.5 mm on the left, both horizontally and vertically.

Refractive error (Retinomax K plus 5 Righton, Japan) was − 15.50 (67 -3.75) in the left eye, while refraction could not be measured in the right eye. Axial length measurement was not possible on the right but was 29 mm (UD-800, Tomey Corporation, Nagoya, Japan) on the left.

The cornea appeared normal. Cryptless iris was observed in both eyes (Fig. 2b, c). Posterior synechiae and a poorly dilated pupil were noted on the right. A white cataract in the right eye obstructed fundus visibility (Fig. 2b). In the left eye, the crystalline lens was clear, and the fundus appearance was myopic with attenuated vessels and attached(Fig. 2d). A distinct tessellated appearance with prominent choroidal vessels was observed on fluorescein angiography (FA)(Fig. 2e). Degenerated vitreous fibers were noticed in the optic disc head and periphery. B-scan ultrasonography (USG) revealed signs of right chronic retinal detachment with thick membrane and funnel configuration (Fig. 2f). On cranial MRI, thinning of the bone thickness was observed in the occipital region (Fig. 2g).

A homozygous c.3502 C > T gene mutation was detected in the COL18A1 gene through whole-exon sequence analysis.

## Discussion

Knobloch syndrome(KNO) is a rare genetic disorder first described by an ophthalmologist William Hunter Knobloch in 1971 [1]. This syndrome follows an autosomal recessive pattern and presents with a group of phenotypic changes, including high myopia, vitreoretinal degeneration (usually with pediatric retinal detachment), and occipital region abnormalities [2].

Knobloch syndrome is associated with mutations in the COL18A1 gene on chromosome 21. However, some cases that could not be associated with any chromosome were also detected and classified as Knobloch syndrome type 2. Knobloch syndrome type 3 is linked to a specific region on chromosome 17 [3]. Both cases discussed in this report are classified as KNO type1.

Suzuki et al. showed that mutations in the COL18A1, consisting of 43 exons, can occur in many different regions, mostly in intron-exon junctions. They demonstrated that these mutations can occur due to frameshift, premature stopping, and splicing. They also reported nonallelic genetic heterogeneity and multiple mutations in KNO [4]. More than 20 possible pathogenic mutations in COL18A1 leading to recessively inherited diseases have been previously reported [4]. Unsurprisingly, genetic analyses of a small number of cases performed due to clinical suspicion resulted in the detection of new mutations. Homozygosity is common in Knobloch syndrome, with reported cased of heterozygosity and compound heterozygosity [3, 4]. In the two cases presented, there were homozygous mutations in the COL18A1 gene on chromosome 21. The genetic result was of unknown clinical significance in the first case (c4376C > T) (pSer1459.Leu). A variant of unknown clinical significance is a DNA change that is not yet fully understood; it may be unique or may have been found in other individuals or families. In the second case, there was a mutation causing premature termination (c.3502 C > T gene mutation). These two mutations should be evaluated together with clinical findings and compared with cases in the literature.

The COL18A1 gene is associated with collagen XVIII synthesis. Collagen type XVIII is found in the epithelial and endothelial basement membranes as a filler. It is expressed throughout the human eye both anterior segment structures (iris, ciliary body, trabecular meshwork, Schlemm canal) and posterior segment structures (the inner limiting membrane, retinal vessels, basement membrane of the retinal pigment epithelium, and Bruch membrane) [4].

Collagen type XVIII plays a critical role in maintaining retinal structure and closing the neural tube [5] and it is part of normal eye development [6]. Changes in the occipital region are one of the striking features of this syndrome, including encephalocele, occipital skin and hair aplasia. The findings were mild in the two reported cases. Even in the MRI imaging, the features could be overlooked. Encephalocele was not observed. The first case was neurologically normal, neurodevelopmental delay was observed in the other case.

Ocular findings include smooth (cryptless) irides, zonular weakness, lens subluxation, pigment dispersion, and associated glaucoma, and some degree of cataract in KNO [7]. Iris transillumination, zonular weakness, lens subluxation, pigment dispersion syndrome, and associated by glaucoma, along with some degree of cataract, are anterior segment findings reported [8]. Anterior segment changes were present in both cases presented in this report. Pupil dilatation, indicating iris anomaly, was poor in both cases. In the first case, iris transullimination defect, tint difference, and posterior embryotoxon were noticeable, accompanied by glaucoma. The posterior synechia in right eye of case 2 might also have contributed to poor dilation.

Similar findings in the two cases were high myopia and high axial length. The inner limiting membrane and vitreous body are known to be important regulators of eye size. Disruption of these structures can cause abnormal growth of the eye. It should be kept in mind that myopia could begin early and progress rapidly in KNO. High myopic error was less in the first case, diagnosed early age. The second case had a higher refractive error and longer axial length.

General retinal findings include peripapillary atrophy, abnormal vitreous condensations, tessellated fundus appearance with prominent choroidal vessels, and macular atrophy in KNO [2]. As in the two cases we presented, retinal detachment (RD) is one of the main findings of the syndrome. KNO is a rare but important cause of pediatric RD, and it should be kept in mind in the differential diagnosis of RD cases presenting with high myopia in newborns and infants.

Information on the type of detachment is limited in the literature. The primary pathophysiology of the accompanying retinal detachment is not clear. One fact we know is that retinal detachment develops in the very early months. Different mechanisms are likely effective in an adult and an individual with normal histoanatomy. Rhegmatogenous or tractional detachment is possible. However, thinning of the retinal layers (the inner limiting membrane, basement membrane of the retinal pigment epithelium, and Bruch membrane) may cause the development of RD without tears. In the presented first case, a hole was not detected, and there were findings of exudative (serous) RD starting from the periphery. The second case had RD complicated with cataracts. The second case had vitreous bands on the retinal surface. No tear was detected in the eye without detachment. The etiology of retinal detachment without tears in CNO cases is still unclear and this statement is based on the author’s opinion. Both cases had asymmetric involvement in both anterior and posterior segments. The effects of the syndrome are more severe in the right eye than the left ones in these presented cases.

Collagen XVIII has an interesting and important property. The C-terminal portion of this collagen is associated with endostatin. Endostatin derives from the C-terminal NC1 domain of this collagen. Endostatin inhibits angiogenesis and can regulate endothelial and neuronal cell migration and proliferation [5, 6, 9]. Suzuki et al. reported that serum endostatin level was variable among KNO cases and might be associated with some systemic phenotypes [4]. The pigmented line observed in the periphery was quite striking in the first case. This line likens to the border of chorioretinal atrophy and detachment developed next to it. We do not know whether the degeneration of the retinal tissue at the border of the pigmented line is related to the effect of any mediator, such as endostatin.

Satisfactory results can be obtained with surgical intervention in first case, but the second one had lost the chance of surgery. The first case late responded to scleral buckling surgery in one eye. A vitrectomy was performed due to non-regressive RD on the other eye. This eye responded well to vitrectomy. Retinal detachment surgery may respond later than expected due to dysgenesis, and additional procedures may be required. Few detachment surgeries have been published in KNO patients. Alsulaiman et al. obtained retinal reattachment with different surgical interventions in 5 eyes of 9 patients [10]. There are prophylactic scleral buckle surgeries in the literature, and they have been reported to be effective [11].

Both scleral buckle and vitrectomy should be on the surgical planning list. Close follow-up is important, and it should be kept in mind that one or more surgeries may be required.

There is phenotypic heterogeneity as well as genetic heterogeneity in KNO. It is unclear how much of each phenotype is actually part of this syndrome, because of the rarity and variability of the condition. Detailed reporting of the phenotypic features of each genetically confirmed case will provide important data to the literature. Despite general developmental and structural dysgenesis, successful anatomical results can be obtained with early surgical interventions. It is essential to recognize the phenotypic and genetic heterogeneity within KNO.

## Data Availability

All data generated during this study are included in this published article and its supplementary information files.

## Abbreviations

KNO: Knobloch syndrome
IOP: intraocular pressure
RD: retinal detachment
MRI: magnetic resonance imagining
USG: ultrasonography
PPV: pars plana vitrectomy
FA: Fluorescein angiography

## References

[1] Knobloch WH, Layer JM (1971). Retinal detachment and encephalocele. J Pediat Ophthalmol.

[2] Khan AO, Aldahmesh MA, Mohamed JY, Al-Mesfer S, Alkuraya FS (2012). The distinct ophthalmic phenotype of Knobloch syndrome in children. Br J Ophthalmol.

[3] Khaliq S, Abid A, White DR, Johnson CA, Ismail M, Khan A et al. Mapping of a novel type III variant of Knobloch syndrome (KNO3) to chromosome 17q11.2. Am J Med Genet A. 2007;143A(23):2768-74. 10.1002/ajmg.a.31739. PMID: 17975799. 10.1002/ajmg.a.31739 17975799

[4] Suzuki OT, Sertié AL, Der Kaloustian VM, Kok F, Carpenter M, Murray J (2002). Molecular Analysis of Collagen XVIII reveals novel mutations, Presence of a third isoform, and possible genetic heterogeneity in Knobloch Syndrome. Am J Hum Genet Volume.

[5] Sertié AL, Sossi V, Camargo AA, Zatz M, Brahe C, Passos-Bueno MR, Collagen XVIII. containing an endogenous inhibitor of angiogenesis and tumor growth, plays a critical role in the maintenance of retinal structure and in neural tube closure (Knobloch syndrome). Hum Mol Genet. 2000;9(13):2051-8. 10.1093/hmg/9.13.2051. PMID: 10942434. 10.1093/hmg/9.13.2051 10942434

[6] Marneros AG, Olsen BR (2005). Physiological role of collagen XVIII and endostatin. FASEB J.

[7] Wawrzynski J, Than J, Gillam M, Foster PJ. Acute angle closure in Knobloch syndrome. J Glaucoma. 2021;30(5):e265-e268. 10.1097/IJG.0000000000001781. PMID: 33449584. 10.1097/IJG.0000000000001781 33449584

[8] Hull S, Arno G, Ku CA, Ge Z, Waseem N (2016). Molecular and clinical findings in patients with Knobloch Syndrome. JAMA Ophthalmol.

[9] O’Reilly MS, Boehm T, Shing Y, Fukai N, Vasios G, Lane WS et al. Endostatin: an endogenous inhibitor of angiogenesis and tumor growth. Cell 88:277–85. 10.1016/s0092-8674(00)81848-6 9008168

[10] Alsulaiman SM, Al-Abdullah AA, Alakeely A, Aldhibi H, Engelbrecht L, Ghazi NG (2020). Macular Hole-related retinal detachment in children with Knobloch Syndrome. Ophthalmol Retina.

[11] Stavros N, Moysidis MD, Hassan A, Aziz MD, Aleksandra V, Rachitskaya MD, Berrocal AM (2014). MD. Prophylactic scleral buckle implantation in Knobloch Syndrome. J Pediat Ophthalmol.

